# Side effects induced by the acute levodopa challenge in Parkinson’s Disease and atypical parkinsonisms

**DOI:** 10.1371/journal.pone.0172145

**Published:** 2017-02-16

**Authors:** Rosario Vasta, Alessandra Nicoletti, Giovanni Mostile, Valeria Dibilio, Giorgia Sciacca, Donatella Contrafatto, Calogero Edoardo Cicero, Loredana Raciti, Antonina Luca, Mario Zappia

**Affiliations:** Department GF Ingrassia, Section of Neurosciences, University of Catania, Catania, Italy; Karolinska Institutet, SWEDEN

## Abstract

**Introduction:**

Acute levodopa challenge may be performed to predict levodopa chronic responsiveness. The aim of the study was to investigate frequency of side effects during the acute levodopa challenge in PD and atypical parkinsonisms.

**Methods:**

We enrolled 34 *de novo* PD patients and 29 patients affected by atypical parkinsonisms (Multiple System Atrophy, MSA, n = 10; Progressive Supranuclear Palsy, PSP, n = 12 and Corticobasal Degeneration, CBD, n = 7) who underwent an acute levodopa challenge. Side effects occurring during test were recorded.

**Results:**

Side effects were more frequent among atypical parkinsonisms as unique group when compared to PD patients (64.3% versus 23.5%; p-value 0.002) with an adjusted OR of 4.36 (95%CI 1.40–13.5). Each atypical parkinsonisms showed almost double occurrence of side effects (MSA 90%, PSP 41.7% and CBD 57%).

**Conclusions:**

Side effects during acute levodopa challenge may be frequent in atypical parkinsonisms. This information could be useful in order to better prepare the patient for the test. Furthermore, it could represent a useful cue in differential diagnosis with PD.

## Introduction

Differential diagnosis between Parkinson’s Disease (PD) and atypical parkinsonisms is still clinical and can be challenging, especially at early stages when clinical features can overlap. Indeed, misdiagnosis has been described in several clinicopathological studies[1].

According to widely accepted clinical diagnostic criteria[2], response to dopaminergic therapy is necessary to support the diagnosis of PD. Dopaminergic responsiveness is usually assessed through chronic levodopa administration even if, in order to predict chronic response, an acute pharmacological test—namely the Acute Levodopa Challenge (ALC)—could be also performed in clinical practice[3,4].

Nevertheless, during ALC it is possible to observe side effects such as nausea, vomiting, confusion, sleepiness, sensation of empty head and general indisposition.

ALC is routinely performed in our Movement disorders center, and during our clinical activity a higher frequency of side effects was observed in atypical parkinsonisms rather than in PD.

Aim of this study was to evaluate the frequency of side effects during ALC in different forms of parkinsonism.

## Materials and methods

### Study design, study population and study period

We retrospectively enrolled all hospitalized patients fulfilling widely accepted clinical diagnostic criteria for Multiple System Atrophy (MSA)[5], Progressive Supranuclear Palsy (PSP)[6] and Corticobasal Degeneration (CBD)[7] at the first visit or during the follow-up, who underwent ALC during the 2010–2014 period. A similar number of patients fulfilling diagnostic criteria for PD[2] at the first visit or during the follow-up, who underwent ALC during the same period were also consecutively enrolled. We enrolled only PD patients never exposed to dopaminergic therapy (i.e. drug-naïve or *de novo*), whereas all patients affected by atypical parkinsonism who already assumed levodopa at the time of the ALC underwent a pharmacological wash-out period. Pharmacological withdrawal methods were in accord to previously adopted study protocols[8,9]. Briefly, medications were gradually withdrawn and worsening of clinical status was assessed daily since patients’ motor condition did not change during two consecutive observational days.

### Acute levodopa challenge

ALC was performed using a standard protocol[8] and administering a single dose of levodopa/carbidopa 250/25 mg. All patients were pre-treated with domperidone 20 mg three times daily for three days, in order to minimize peripheral dopaminergic side effects. Motor response to levodopa was quantified using the Motor Evaluation section of the Unified Parkinson’s Disease Rating Scale (UDPRS-ME)[10]. During the ALC, motor examination was performed immediately before and every hour after the drug intake until the motor conditions returned to the motor baseline status. Amplitude of motor response was calculated as the difference in percentage between the baseline and the peak-of-dose motor scores[8,9]. Side effects during the test were systematically investigated and recorded in an *ad hoc* format. In particular, the following side effects have been considered: nausea, vomiting, sickness, empty head, confusion, sleepiness, asthenia, dizziness, headache and others less common such as sweating, blurred vision, visual hallucinations and anxiety.

Demographic and clinical characteristics have also been recorded. The study was approved by the local ethical committee (Azienda Ospedaliero-Universitaria Policlinico-Vittorio Emanuele, Catania). It is solely based on clinical information related to the diagnostic workup of the study patients at the time of their admission to our clinic; a written informed consent was obtained from the patients.

### Statistical analysis

Quantitative variables were described using mean and standard deviation. The difference between means and the difference between proportions was evaluated by the t-test and the Chi-square test respectively. In case of not a normal distribution appropriate non-parametric tests were performed. In order to identify variables associated with the occurrence of side effects, a logistic regression model was performed considering the presence of side effect during ALC as outcome. Diagnosis of PD versus atypical parkinsonisms, sex, age at onset, age at the ALC, disease duration, UPDRS-ME at baseline, HY and the amplitude of motor response were considered as independent variables. Parameters associated with the outcome at the univariate analysis with a threshold of p-value < 0.10 were included in the model. The model was manually constructed using the likelihood ratio test (LRT) to compare the log-likelihood of the model with and without a specific variable. P-value < 0.05 was considered as statistically significant. Odds Ratio (OR) together with ninety-five percent confidence intervals (95% CI) were calculated assuming the Poisson distribution. Statistical analysis was performed using STATA 12.0 software (StataCorp., College Station, TX, USA).

## Results

Thirty-four patients affected by PD and 29 affected by atypical parkinsonisms (10 MSA, 12 PSP and 7 CBD) were eligible for the study. Thirteen patients with atypical parkinsonisms were untreated at the time of the study, while 16 were in levodopa therapy and underwent a pharmacological wash-out before the ALC. No differences were found in demographic characteristics between the two groups, while, as expected, patients affected by atypical parkinsonisms showed higher scores in both UPDRS-ME and Hoehn and Yahr scale (Table 1). PD patients showed significant higher amplitude of motor response with respect to atypical parkinsonisms (7.9 ± 0.8 *versus* 2.5 ± 5.8 respectively; p-value 0.02).

**Table 1:** Baseline characteristics of patients affected by PD and atypical parkinsonisms.

	PD (n = 34)	MSA (n = 10)	PSP (n = 12)	CBD (n = 7)	Atypical parkinsonisms (n = 29)	p-value*
Men (%)	15 (44.1%)	3 (30.0%)	7 (58.3%)	5 (71.4%)	15 (51.7%)	0.6
Age at onset (years)	60 ± 10.2	56.9 ± 6.9	63.8 ± 5.6	70.3 ± 2.6	63 ± 7.5	0.2
Disease duration (years)	1.9 ± 2.7	2.3 ± 1.7	3.3 ± 1.3	2.9 ± 2.5	2.8 ± 1.8	0.1
UPDRS-ME baseline	24.8 ±11.7	29.0 ± 16.3	32.0 ± 9.0	37.4 ± 15.8	32.3 ± 12.5	**0.02**
Hoehn-Yahr stage	1.8 ± 0.6	2.5 ± 1.0	2.7 ± 0.7	2.4 ± 0.5	2.6 ± 0.8	**0.0001**
Amplitude of motor response to ALC**	7.9 ± 10.8	1.7 ± 4.5	0.9 ± 2.2	6.5 ± 9.6	2.5 ± 5.8	**0.02**
Side effects during ALC	8 (23.5%)	9 (90.0%)	5 (41.7%)	4 (57.1%)	18 (62.1%)	**0.002**

ALC = acute levodopa challenge; amplitude of motor response to levodopa was calculated as the difference in percentage between the baseline and the peak-of-dose UPDRS-ME scores.

* P-value refers to the comparison between PD and atypical parkinsonisms considered as a unique group.

** Difference in percentage between the baseline and the peak-of-dose UPDRS-ME scores after the levodopa administration.

Side effects during ALC were significantly more frequent among atypical parkinsonisms, considered as a unique group, with respect to PD patients (62.1% *versus* 23.5%; p-value 0.002). MSA presented the highest frequency of side effects occurring in 9 patients (90%), followed by CBD in 5 patients (71.4%) and PSP in 4 patients (33.3%). A significant difference with respect to the frequency recorded among PD patients (23.5%) was achieved only for MSA (p-value < 0.001). The most frequent side effect was nausea, occurring in a total of 11 (17.5%) patients (Table 2).

**Table 2:** Frequency of different side effects in PD and atypical parkinsonisms.

	PD (n = 34)	MSA (n = 10)	PSP (n = 12)	CBD (n = 7)	Atypical parkinsonisms (n = 29)
Nausea	2 (5.9%)	3 (30%)	2 (16.7%)	4 (57.1%)	9 (31.0%)
Vomiting	0	2 (20%)	0	0	2 (6.9%)
Sickness	1 (2.9%)	2 (20%)	0	1 (14.3%)	3 (10.3%)
Empty head	1 (2.9%)	0	0	1 (14.3%)	1 (3.5%)
Confusion	1 (2.9%)	2 (20%)	0	0	2 (6.9%)
Sleepiness	3 (8.8%)	1 (10%)	2 (16.7%)	1 (14.3%)	4 (13.8%)
Asthenia	0	2 (20%)	0	0	2 (6.9%)
Dizziness	0	2 (20%)	0	2 (28.6%)	4 (13.8%)
Headache	0	0	0	3 (42.9%)	3 (10.3%)
Others	2 (5.9%)	0	0	2 (28.6%)	2 (6.9%)
At least one side effect	8 (23.5%)	9 (90%)	4 (33.3%)	5 (71.4%)	18 (62.1%)

PD = Parkinson’s Disease; MSA = Multiple System Atrophy; PSP = Progressive Supranuclear Palsy; CBD = Corticobasal Degeneration; others = sweating, blurred vision, visual hallucinations or anxiety.

At univariate analysis atypical parkinsonisms, considered as a unique group, were significantly associated with the presence of side effects during ALC with an OR of 5.31 (95% CI, 1.78–15.8; p-value 0.003), while the amplitude of motor response showed a borderline significant negative association (OR 0.93; 95%CI 0.86–1.01; p-value 0.07). No association was found for sex, age at onset, age at the ALC, disease duration, UPDRS-ME and HY. The positive association between atypical parkinsonisms and side effects was still significant after adjusting by the amplitude of motor response at the multivariate analysis (adjusted OR 4.36; 95% CI, 1.40–13.5; p-value 0.01).

## Discussion

ALC may be performed in order to predict levodopa chronic responsiveness and hence to help differentiating PD patients from patients affected by other parkinsonisms. Currently, PD diagnosis is clinical, assessed through diagnostic criteria whose accuracy ranges from 75% to 95%, depending on measures of accuracy adopted, age, disease duration and the expertise of the clinician[11]. Indeed, especially at early stages, PD can share clinical features with other parkinsonisms, particularly with MSA and PSP, and this can lead to misdiagnosis.

In our sample we found a suboptimal motor response at the ALC in PD patients. According to a previous study, amplitude of motor response may be suboptimal at the ALC in *de novo* PD patients who demonstrate a long-term levodopa responsiveness, showing an increment over time during chronic treatment [8]. On the other hand, a positive acute response to levodopa may be not specific for PD, being also observed in atypical parkinsonisms, particularly in MSA[12].

We evaluated the frequency of side effects during the ALC in PD and in different form of atypical parkinsonisms. To the best of our knowledge, only one study has explored the possible role of clinical intolerance to ALC in differentiating MSA from PD. Authors reported a higher even if not significant frequency of side effect among patients affected by MSA with respect to PD patients (80% and 55.9% respectively). Nonetheless, the small size of the samples (a total of 5 MSA) limits the interpretation of the results.[13]

In our sample atypical parkinsonisms showed a significant five fold increased risk to develop side effects during ALC. Demographic and clinical characteristics were not significantly associated with the presence of side effect with the exception, as expected, of the amplitude of motor response that showed a borderline significant negative association at univariate analysis. Considering the different form of atypical parkinsonisms, MSA showed the highest frequency of side effect during the levodopa test (90%); incidence of side effects among PSP and CBD was about double respect to PD patients, even if such a difference was not significant probably due to a lack of power related to the low number of cases.

We have not a clear explanation for the higher frequency of side effects among atypical parkinsonisms. The possible widespread degeneration involving different systems among atypical parkinsonisms or in some extent the higher responsiveness to levodopa could probably play a role, but, to the best of our knowledge, no experimental studies on animal model are available in literature in order to provide evidence that could explain this observation. We are aware that the lack of plasmatic levodopa concentrations during the test could represent a limit in the interpretation of results. Since it has been shown that plasmatic levodopa levels do not correlate with efficacy on motor symptoms[9], it could be hypothesized that neither the occurrence of side effects does. At any rate, a different levodopa pharmacokynetics in atypical parkinsonisms cannot be excluded.

In conclusion, we believe that the more frequent occurrence of side effects during ALC among atypical parkinsonisms observed in our study could represent an useful information in order to better prepare the patient to the test. Indeed, when atypical parkinsonism is suspected, precautions such as a more proper antiemetic pre-treatment or prevention of orthostatic hypotension could be taken, even if the lack of systematic information regarding chronic levodopa assumption did not allow us to assess whether the occurrence of side effects during ALC could predict chronic intolerance to levodopa. Furthermore, our results could represent an additional cue in the differential diagnosis of parkinsonisms. However, the small sample size of the study, mainly related to the low incidence of atypical parkinsonisms in the population, and the lack of a definitive autoptic diagnosis constitute important limits in the interpretation of the results. Future larger and multicenter studies are needed in order to better investigate this hypothesis.
